# Broadband terahertz metamaterial absorber based on sectional asymmetric structures

**DOI:** 10.1038/srep32466

**Published:** 2016-08-30

**Authors:** Cheng Gong, Mingzhou Zhan, Jing Yang, Zhigang Wang, Haitao Liu, Yuejin Zhao, Weiwei Liu

**Affiliations:** 1 Institute of Modern Optics, Nankai University, Key Laboratory of Optical Information Science and Technology, Ministry of Education, Tianjin 300071, China; 2 School of Electronic Engineering, University of Electronic Science and Technology of China, Chengdu, 611731, China; 3 School of Optoelectronics, Beijing Institute of Technology, Beijing, 100081, China

## Abstract

We suggest and demonstrate the concept and design of sectional asymmetric structures which can manipulate the metamaterial absorber’s working bandwidth with maintaining the other inherent advantages. As an example, a broadband terahertz perfect absorber is designed to confirm its effectiveness. The absorber’s each cell integrates four sectional asymmetric rings, and the entire structure composed of Au and Si_3_N_4_ is only 1.9 μm thick. The simulation results show the bandwidth with absorptivity being larger than 90% is extended by about 2.8 times comparing with the conventional square ring absorber. The composable small cell, ultra-thin, and broadband absorption with polarization and incident angle insensitivity will make the absorber suitable for the applications of focal plane array terahertz imaging.

In 2011, Tao *et al.* from Boston University proposed a metamaterial absorbing enhanced optical-readout bimaterial cantilever pixels for Microwave and Terahertz (THz) wave imaging^1^. Since then, the bimaterial cantilever focal plane array (FPA) imaging sensor integrated with metamaterial absorber has gained increased attention owing to its many distinctive properties^2^,^3^,^4^,^5^. In 2012, Alves *et al.* reported a micro-electro-mechanical systems (MEMS) bimaterial THz sensor operating at 3.8 THz. Its pixels integrated metamaterial absorber and the measurement showed that the fabricated absorber has nearly 90% absorption at 3.8 THz^2^,^3^. In 2013 and 2014, they improved their design and the absorption achieved near 100% at 3.8 THz and the responsivity is around 1.2 deg/μW^4^,^5^. In 2015, Ma *et al.* demonstrated an infrared bimaterial cantilever focal plane array integrated with metamaterial absorber to enhance the infrared imaging performance^6^.

The metamaterial absorbers provide unprecedented ability to absorb energy of electromagnetic waves. The absorbed energy heats bimaterial cantilever to deform its optical-readout structure. To increase the sensitivity and reduce the response time of the cantilever sensor, relatively thin absorber which is compatible with MEMS-based bimaterial fabrication process is required^3^. To improve the imaging resolution, the sensor’s unit cell should be small enough^4^. To enhancing dynamic range of the deformable cantilever sensor, the broadband absorption with polarization insensitivity and incident angle insensitivity is important. However, it is a great challenge to simultaneously meet all the aforementioned properties (ultra-thin, small cell, and broadband), especially in the THz frequency range.

Since Padilla *et al* demonstrated the metamaterial absorber in 2008^7^,^8^, many groups began to study high performance broadband THz metamaterial absorbers^9^,^10^,^11^,^12^,^13^,^14^,^15^,^16^,^17^,^18^,^19^,^20^,^21^,^22^,^23^,^24^. In general, there are several methods are widely used for extending the bandwidth. The first is combing multiple metamaterial structures (or cells) with approximate resonance frequencies into a large cell^9^,^10^,^11^. The second is nesting multiple metamaterial structures (or cells) into a new cell^12^,^13^,^14^. The third is stacking multiple structures into a new multilayer cell^15^,^16^,^17^,^18^,^19^,^20^. The fourth is based on doped silicon grating^21^,^22^,^23^,^24^. However, the first method will make the absorber’s unit cell too large. The second, third and fourth methods are constrained by the structures’ spatial arrangement, fabrication process, or thickness.

To overcome the limitations of prior works, we suggest the concept of sectional asymmetric metamaterial structures which can manipulate specific device’s working bandwidth with maintaining the inherent advantages of the device. In the following a broadband THz absorber based on the structures will be demonstrate to confirm this idea. The absorber integrates four sectional resonators into a small cell with inheriting the complete resonator’s advantages of polarization insensitivity and incident angle insensitivity, while the bandwidth with absorptivity being larger than 90% is extended to about 2.8 times. Its entire structure which is compatible with MEMS-based fabrication is only 1.9 μm thick. The small cell, ultra-thin, and broadband absorption will make the absorber suitable for the applications of optical-readout bimaterial cantilever focal plane array imaging sensor^4^,^5^, or some electrical-readout imaging sensors such as microbolometer sensor^25^,^26^,^27^ and pyroelectric sensor^28^.

## Principle and Design

**Figure 1:**
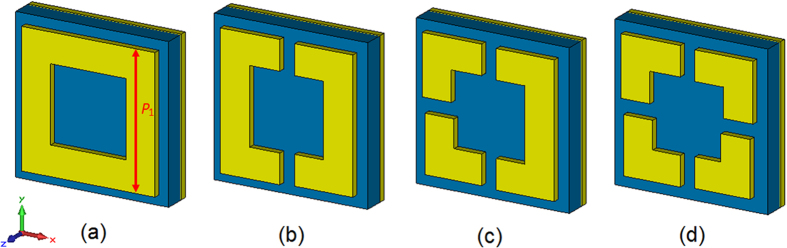
Design example of a sectional asymmetric metamaterial unit cell. (**a**) The basic structure: complete square ring in one cell and its outer side length is *P*_1_. Then, dividing the symmetrical structure into sections and varying the characteristic size of each section: (**b**) two asymmetric sectional square rings and (**c**) three asymmetric sectional square rings. Finally, compose these asymmetric sections into a new unit cell: (**d**) the final sectional asymmetric square ring cell.

The basic idea of sectional asymmetric structures can be explained in such a simple way that multiple sectional structures in one cell result in coupling of multiple resonant frequencies. It will extend the bandwidth effectively by combing the frequency responses. As an example, Fig. 1 describes the design process of a sectional asymmetric cell. First of all, choosing one complete metamaterial structure as basic structure as is shown in Fig. 1(a). And then, dividing the symmetrical structure into sections and varying the characteristic size of each section as shown in Fig. 1(b,c). Finally, compose these asymmetric sections into a new unit cell and the cell is called sectional asymmetric cell which is shown in Fig. 1(d).

**Figure 2:**
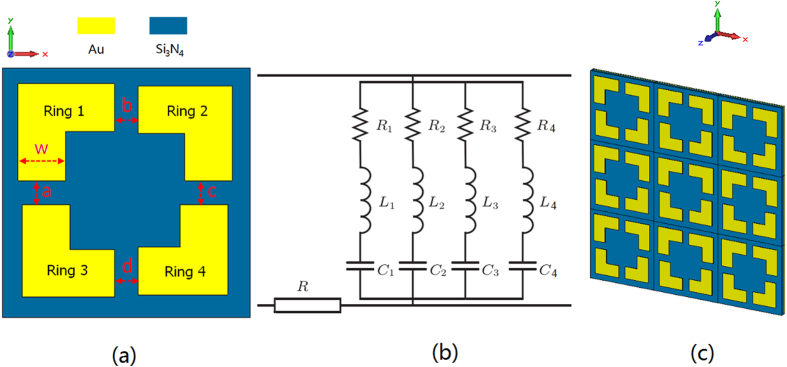
Proposed broadband THz sectional asymmetric metamaterial absorber. (**a**) Top structure in one cell of the absorber. (**b**) Transmission line model of the cell. (**c**) Diagrammatic sketch of a 3 × 3 cells array.

Next, we proposed a broadband THz absorber based on the above example to verify its effectiveness. The basic structure is a simple square ring resonator which has advantages of polarization insensitivity and incident incidence angle insensitivity^12^. Figure 2(a) describes one cell of the absorber. Figure 2(c) shows a 3 × 3 cells’ array. The cell includes four quarter sectional square rings: Ring 1, Ring 2, Ring 3, and Ring 4, and these rings form an asymmetric ring structure. The outer side length of four complete square rings are *P*_1_ = 19.4 μm, *P*_2_ = 19 μm, *P*_3_ = 18.4 μm, and *P*_4_ = 18 μm, respectively. The gaps a, b, c, and d between the sectional rings are 2.2 μm and the width of the sectional ring is w = 3.8 μm. The period of one metamaterial cell is 22 μm.

The absorber is made of Au and Si_3_N_4_ which are compatible with MEMS-based fabrication. It comprises three layers: the top layer is an array of sectional asymmetric Au resonators (0.1 μm thick); the middle layer is Si_3_N_4_ dielectric film (*t* = 1.7 μm thick); the bottom layer is Au ground (0.1 μm thick). The thermal, mechanical, and optical properties of Au and Si_3_N_4_ make the absorber suitable for applications of bimaterial cantilever focal plane array optical readout imaging^29^.

The physical concepts and explanation of the bandwidth broadening are as follows: 1) To realize perfect absorption the impedance of the absorber should be matched to that of free space, for example: μ = ε then Z = (μ/ε) ^1/2^ = 1, which ε stands for the normalized electric permittivity, μ is the normalized magnetic permeability, and Z represents the normalized free space impedance. The sectional square ring (Au bracket) is an example of electric ring resonator (ERR) and couples strongly to uniform electric fields, but negligibly to a magnetic field. By adding a ground plane, the magnetic component of the incident electromagnetic wave induces a current in the sections of the ERR that are parallel to the direction of the E-filed. The electric and magnetic response can then be tuned independently. The ERR determines the electric response while the dielectric type and thickness between the ERR and the ground plane determines the magnetic response. 2) In this design, the resonant frequency of the absorption peak is mainly determined by the outer side length *P* of basic square rings. Therefore, we integrate four sectional square rings with different size (*P*_*1*_*–P*_*4*_) to support multiple resonant modes closely positioned together in the absorption spectrum. By tuning the thickness of the dielectric film, the sectional asymmetric structure can be impedance-matched to free space at each resonant frequency and absorption broadening is achieved.

To provide a further interpretation, the Transmission line model of the sectional asymmetric metamaterial cell is described in Fig. 2(b). *R* stands for electromagnetic loss of the Si_3_N_4_ dielectric film. The four sectional square rings are equivalent to four parallel RLC circuit. *R*_1_, *R*_2_, *R*_3_, *R*_4_ represent ohm resistance of the four Au sectional rings. *L*_1_, *L*_2_, *L*_3_, *L*_4_ stand for equivalent inductance and *C*_1_, *C*_2_, *C*_3_, *C*_4_ are equivalent capacitance of the four sectional rings, respectively. According to the RLC circuit model, the resistance will not affect the resonance frequency, so we omitted it for simplicity. The inductance and capacitance can approximately describe the resonance of sectional asymmetric structures. The inductance of each sectional square ring can approximately be given by *L*_*i*_ ~ (*t*/*w*) · *P*_*i*_, where *t* is thickness of the Si_3_N_4_ dielectric film, *w* is the width of the sectional ring, *P*_*i*_ is the outer side length of the basic square ring. The capacitance can be expressed by *C*_*i*_ ~ (*P*_*i*_ · *w*)/*t*. Then the resonance frequency of the specific sectional ring is given by^30^,^31^:


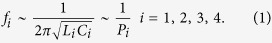


Here *i* represent the sectional ring resonators. The Equation 1 demonstrates that the resonance frequency *f* is an approximate linear function of 1/*P*_*i*_. Therefore, four sectional square rings will correspond to four resonance frequencies. The bandwidth of the absorber will be broadened by optimizing and adjusting the four outer side lengths *P*_1_, *P*_2_, *P*_3_, and *P*_4_.

## Results and Discussions

The proposed absorber can be regarded as an effective media and characterized by a complex electric permittivity ε and complex magnetic permeability μ. The resonance structures couple strongly to the electric or magnetic fields and match the impedance Z = (μ/ε)^1/2^ to free space to minimize the reflectance. The absorber’s reflectance and transmission can be acquired by simulating the complex frequency dependent S parameters, S_11_ and S_21_^7^. Then, the absorptivity *A* is calculated by


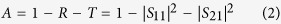


where *R* = |S_11_|^2^ and *T* = |S_21_|^2^ are the reflectance and transmission, respectively. In our design the transmission *T* is zero because of the Au ground. Therefore, the absorptivity can be given by *A = *1*−R.*

**Figure 3:**
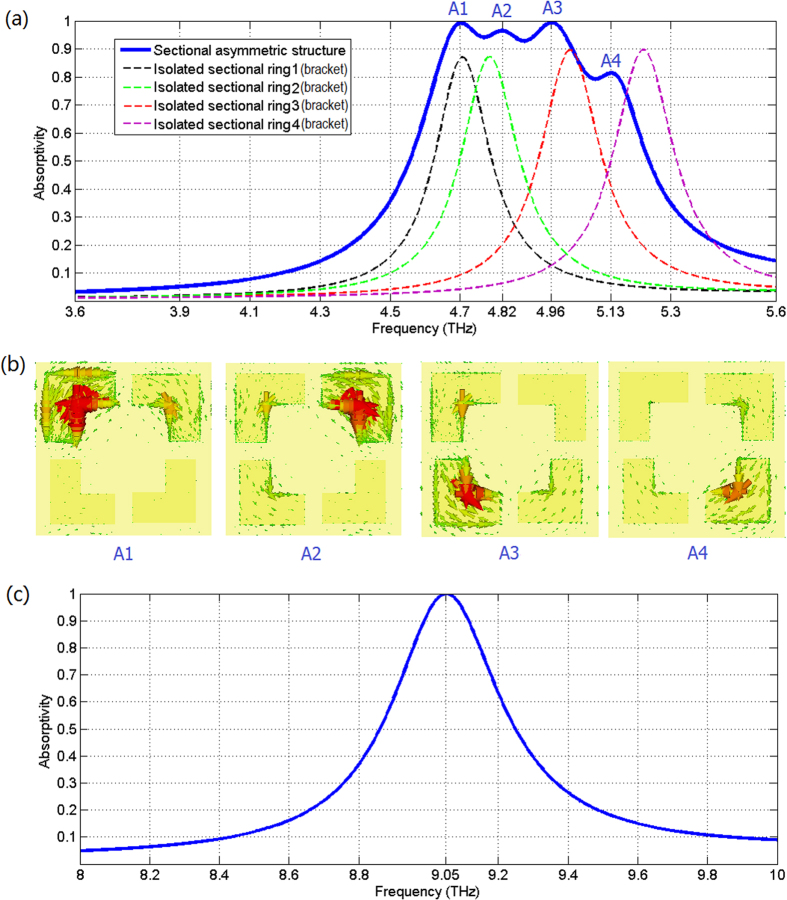
Simulation results. (**a**) Absorptivity curve of the sectional asymmetric metamaterial absorber; for comparison the absorption spectra of isolated Au brackets (quarter sectional ring) absorber are also provided. (**b**) Resonance current distributions in one cell at different absorption peaks. (**c**) Absorptivity curve of the compared metamaterial absorber based on complete square ring.

We modeled the sectional asymmetric structures and simulated the electromagnetic characteristics by the commercial 3D electromagnetic simulator CST Studio Suite 2012. The thickness of its Si_3_N_4_ dielectric film is 1.7 μm and the dielectric constant is 8. The material of the sectional ring is Au with an electric conductivity 4.6 × 10^7^ s/m and the thickness of the ring is 0.1 μm. The unit cell boundary condition with Floquet-port was used to simulate the absorption spectra, because the Floquet mode can be applied to any periodical array whether mirrored or rotated. The absorptivity curve of sectional asymmetric absorber is shown in Fig. 3(a) and there are four absorption peaks A1 = 99.2%, A2 = 96.4%, A3 = 99.3%, and A4 = 81.3% corresponding to four resonance frequencies 4.7 THz, 4.82 THz, 4.96 THz, and 5.13 THz, respectively. The bandwidth with absorptivity being larger than 90% is about 364 GHz. Figure 3(b) is the resonance current distributions in one cell at different absorption peaks. It shows that the sectional square ring1 primarily contributes to absorption peak A1, the ring2 contributes to absorption peak A2, the contributions of ring3 and ring4 corresponding to absorption peak A3 and A4, respectively. Moreover, we provide the absorption spectrum in the situation of only having isolated Au bracket (quarter sectional ring) in Fig. 3(a). The four absorption spectra are all narrowband and the absorption peaks are located at 4.706 THz, 4.784 THz, 5.014 THz, and 5.222 THz respectively. It’s worth mentioning that the maximum absorptivity of the isolated Au bracket absorber is only about 90% and lower than the counterpart (about 99%) of their combination. The results demonstrate that the sectional asymmetric structure can realize better impedance-matched to free space by the coupling of four isolated Au brackets effectively.

Furthermore, as a comparison we modeled an absorber based on a complete square ring structure. The period of its unit cell is 22 μm and the outer side length of the square ring is *P*_4_ = 18 μm. The layer and materials are the same as the proposed sectional asymmetric broadband absorber. Figure 3(c) shows its absorptivity curve. The bandwidth with absorptivity being larger than 90% is only about 126 GHz. The bandwidth of sectional asymmetric structure absorber is about 364/126 = 2.8 times than that of complete square ring structure absorber.

**Figure 4:**
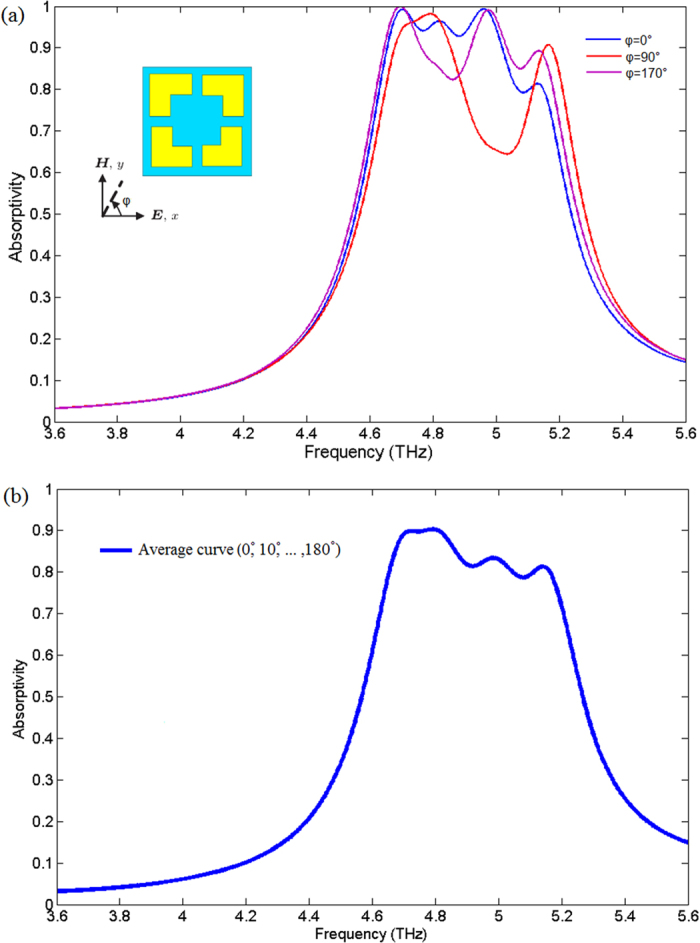
Simulation and calculation results. (**a**) Absorptivity curves at various polarization angles φ for the normal incident radiation. The polarization angle φ is defined as the angle between electric field E and positive *x* axis and depicted as the inset. (**b**) The calculated average curve of these absorptivity curves.

In application of focal plane array THz imaging, the sensor integrated absorber should be insensitive to the incidence angles, especially for the optical-readout cantilever sensor which will deflect angles owing to the bimaterial thermal effect, and the absorber should be polarization insensitive to maximize the absorption. To evaluate these characteristics, we simulated the absorption spectra of sectional asymmetric broadband absorber in different polarization angles and incidence angles by the CST2012. Figure 4(a) shows the absorptivity curves at various polarization angles for the normal incident radiation. Figure 4(b) depicts the calculated average curve of the absorptivities according to the various polarization angles. The polarization angle φ is defined as the angle between electric field E and positive *x* axis and depicted as the inset in Fig. 4(b). The equation for calculating average absorption curve of Fig. 4(b) is given by


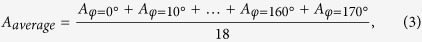


where *A*_*average*_ stands for the average absorptivity at the different polarization angles, *A*_φ_ = 0°, *A*_φ_ = 10°, …,*A*_φ_ = 160°, *A*_φ_ = 170° represents the absorptivity at polarization angles *φ* = 0°, *φ* = 10°, …,*φ* = 160°, *φ* = 170°, respectively.

The major metrics to define polarization-insensitive are 1) the absorptivity; 2) the resonance frequency. The absorbers which are sensitive to polarization will have severer absorptivity decline and resonance frequency shift^7^ in different polarization directions. For the polarization-insensitive absorbers, although they cannot realize uniform absorption exactly at different polarizations and most of polarization-insensitive absorbers will show differences between neighboring polarizations, but the polarization-insensitive absorbers can keep relative high absorption and the resonance frequency will not be offset too much in different polarization directions. As is shown in Fig. 4, although the absorptivity has fluctuation when φ sweeps from 0° to 170°, the simulation results show that the proposed structure provides relative high absorptivity (The range of values is about 60~99%) and keeps broadband absorptions (resonance frequency almost no shift) at different polarization angles. Furthermore, the calculated average absorption according to the polarization angles (φ = 0°, φ = 10°, …. φ = 160°, φ = 170°) also shows broadband high absorption in the same frequency range.

**Figure 5:**
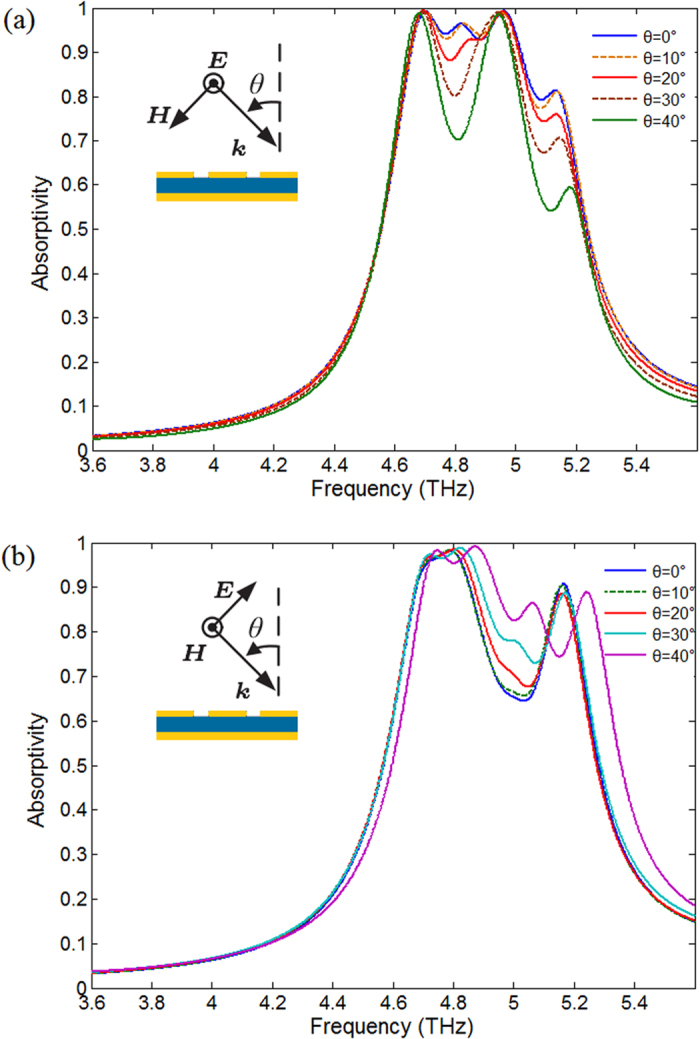
Simulation results. (**a**) Absorptivity curves at various oblique incident angle θ for the TE radiation. (**b**) Absorptivity curves at various oblique incident angle θ for the TM radiation. The incidence angle θ is defined as the angle between wave vector *k* and the surface normal of the absorber and is depicted as the insets.

Figure 5(a) describes how the absorptivity changes for different incidence angles which increase with a step of 10 degrees from 0° to 40° for the TE (Transverse Electric) radiation. Figure 5(b) shows how the absorptivity changes for the TM (Transverse Magnetic) radiation. The incidence angle θ is defined as the angle between wave vector *k* and the surface normal of the absorber and is depicted as the insets.

According to the Fig. 5, the absorptivity decreases a little when φ sweeps from 0° to 40° for the TE wave. Meanwhile, the absorptivity increases and has frequency shift when φ sweeps from 0° to 40° for the TM wave. Although the absorptivity has fluctuation, the simulation results demonstrate that the absorber keeps the characteristic of broadband absorption at various oblique incident angles.

## Conclusion

The paper proposed the concept of sectional asymmetric structures. They can manipulate specific device’s working bandwidth with maintaining the properties of the basic structure. To verify its effectiveness a broadband THz absorber was designed and evaluated numerically. Each cell of the absorber integrates four sectional square rings with approximate resonance frequencies. The entire structure composed of Au and Si_3_N_4_ is only 1.9 μm thick. The simulation results show its bandwidth with absorptivity being larger than 90% is extended to about 2.8 times comparing with the classic square ring absorber.

We believe the sectional asymmetric structures have two distinctive advantages: 1) manipulating specific device’s working bandwidth while maintaining the inherent advantages of the complete structure; 2) not increasing size of the structure and thickness of the device. Therefore, it will be suitable for the focal plane array THz imaging sensors such as optical-readout bimaterial cantilever sensors or some electrical-readout sensors (microbolometer sensors and pyroelectric sensors).

## Methods

### Design of the sectional asymmetric metamaterial structures

There are four steps to design a normal sectional asymmetric structure (or cell). The first step is choosing one complete resonance structure as basic structure. The second step is dividing the symmetrical structure into sections. The third step is varying the characteristic size of each section. The fourth step is composing these asymmetric sections into a new structure (cell). Then, we called it sectional asymmetric metamaterial structure. It should be mentioned that the selected basic structure in the first step should be multiple symmetric structures, such as square ring, hexagonal ring, circular ring, or elliptical ring and so on.

**Figure 6:**
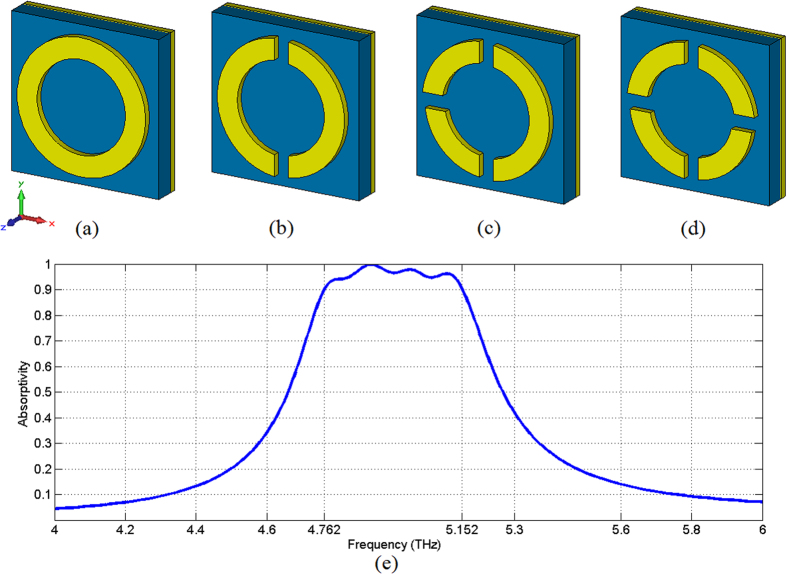
Design example of the sectional asymmetric metamaterial structure. (**a**) The basic structure: complete circle ring in one cell. (**b**) Then, dividing the symmetrical structure into sections and varying the characteristic size of each section: (**b**) two asymmetric sectional circle rings and (**c**) three asymmetric sectional circle rings. (**d**) The final sectional asymmetric circle ring cell. (**e**) Absorptivity curve of the designed metamaterial absorber simulated by CST 2012.

We take another simple example to demonstrate the design method. This example is also a metamaterial absorber made of Au and Si_3_N_4,_ but its basic structure is circular ring. The absorber comprises three layers: the top layer is an array of sectional asymmetric Au resonators (0.1 μm thick); the middle layer is Si_3_N_4_ dielectric film (1.8 μm thick); the bottom layer is Au ground (0.1 μm thick). Figure 6(a) shows one cell based on a complete circular ring. After the aforementioned four steps, the Fig. 6(d) describes the final sectional asymmetric unit cell which integrates four circle sections with four resonance frequencies. The outer radiuses of four circle rings are 9.6 μm, 9.4 μm, 9.2 μm, and 9 μm, respectively. The gaps between the sectional rings are 2.1 μm and the width of the sectional circle ring is 3 μm. The period of one metamaterial cell is the same as the proposed sectional asymmetric square ring absorber (22 μm). Figure 1(e) depicts the absorptivity curve of the metamaterial absorber simulated by CST 2012. The absorber’s bandwidth with absorptivity being larger than 90% is about 390 GHz, but its entire thickness is only 2 μm. In addition it’s worth mentioning that we can broaden the absorption spectra using more sectional rings, or narrow the absorption spectra using fewer sectional rings.

### Numerical simulation

We modeled the cell structures and simulated their electromagnetic characteristics by the commercial 3D electromagnetic simulator CST Studio Suite 2012. We use auto meshing technology and frequency-domain solver to acquire S-parameter. The unit cell boundary condition with Floquet-port was used to simulate the absorption spectra, because the Floquet mode can be applied to any periodical array whether mirrored or rotated. The resonance current distributions were obtained based on the Field Monitor technology of the CST at certain frequencies. The thickness of Si_3_N_4_ dielectric film is 1.7 μm and its dielectric constant is 8. The material of the sectional ring is Au with an electric conductivity 4.6 × 10^7^ s/m and the thickness of the ring is 0.1 μm.

## Additional Information

**How to cite this article**: Gong, C. *et al.* Broadband terahertz metamaterial absorber based on sectional asymmetric structures. *Sci. Rep.* **6**, 32466; doi: 10.1038/srep32466 (2016).
